# Alterations in metabolic flux in migraine and the translational relevance

**DOI:** 10.1186/s10194-022-01494-w

**Published:** 2022-09-30

**Authors:** Olivia Grech, Matilde Sassani, Gisela Terwindt, Gareth G. Lavery, Susan P. Mollan, Alexandra J. Sinclair

**Affiliations:** 1 Metabolic Neurology, Institute of Metabolism and Systems Research, College of Medical and Dental Sciences, University of Birmingham, Edgbaston, Birmingham, B15 2TT UK; 2 Department of Neurology, University Hospitals Birmingham NHS Foundation Trust, Birmingham, B15 2GW UK; 3 Department of Neurology, Leiden University Medical Center, Leiden, The Netherlands 2333 ZA; 4 Department of Biosciences, School of Science and Technology, Nottingham Trent University, Clifton Campus, Nottingham, NG11 8NS UK; 5 Birmingham Neuro-Ophthalmology, Queen Elizabeth Hospital, University Hospitals Birmingham, Birmingham, B15 2GW UK

**Keywords:** Headache, Migraine, Metabolism, CGRP, Metabolic disorders, Nutraceuticals, Obesity, Glucose metabolism

## Abstract

**Background:**

Migraine is a highly prevalent disorder with significant economical and personal burden. Despite the development of effective therapeutics, the causes which precipitate migraine attacks remain elusive. Clinical studies have highlighted altered metabolic flux and mitochondrial function in patients. In vivo animal experiments can allude to the metabolic mechanisms which may underlie migraine susceptibility. Understanding the translational relevance of these studies are important to identifying triggers, biomarkers and therapeutic targets in migraine.

**Main body:**

Functional imaging studies have suggested that migraineurs feature metabolic syndrome, exhibiting hallmark features including upregulated oxidative phosphorylation yet depleted available free energy. Glucose hypometabolism is also evident in migraine patients and can lead to altered neuronal hyperexcitability such as the incidence of cortical spreading depression (CSD). The association between obesity and increased risk, frequency and worse prognosis of migraine also highlights lipid dysregulation in migraine pathology. Calcitonin gene related peptide (CGRP) has demonstrated an important role in sensitisation and nociception in headache, however its role in metabolic regulation in connection with migraine has not been thoroughly explored. Whether impaired metabolic function leads to increased release of peptides such as CGRP or excessive nociception leads to altered flux is yet unknown.

**Conclusion:**

Migraine susceptibility may be underpinned by impaired metabolism resulting in depleted energy stores and altered neuronal function. This review discusses both clinical and in vivo studies which provide evidence of altered metabolic flux which contribute toward pathophysiology. It also reviews the translational relevance of animal studies in identifying targets of biomarker or therapeutic development.

## Background

Migraine is a highly prevalent and disabling disorder, affecting over 1 billion people worldwide [1] and ranking the leading cause of disability amongst neurological conditions [2]. In addition to economical loss, migraine significantly reduces quality of life, [3] and is often comorbid with stress, anxiety and depression [4]. In recent years there has been development of effective migraine therapeutics which target the release of the nociceptive neuropeptide, calcitonin gene related peptide (CGRP). Although these agents are effective at reducing migraine days and improving quality of life [5], the factors which contribute towards the precipitation of headache attack in those with migraine remain unclear.

Migraine is a prevalent feature of both mitochondrial and metabolic disorders (lifetime prevalence of 61% for mitochondrial [6] and 1-year prevalence of 11.9% in men and 22.5% in women with metabolic disorders [7]), which suggests migraine may be a common clinical manifestation of brain energy dysfunction [8]. Moreover, advancement in metabolic imaging and monitoring techniques has led to the hypothesis that the metabolic flux is altered in migraine patients, [9] leading to an energetic deficit which could underlie headache susceptibility. This review aims to evaluate animal and clinical studies which provide evidence of the perturbations in metabolic flux in migraine. Furthermore, it will discuss the translational relevance of these studies and identify altered energetic pathways which may serve as biomarkers or targets for therapeutic development.

**Table 1:** Summary of the evidence of perturbed metabolic pathways from patient studies and in vivo data, and their hypothesised implications in migraine pathophysiology

Metabolic pathway	Evidence of alterations in patients	Evidence of alterations in animal models	Potential implications in migraine pathophysiology
Glucose utilization	• Glucose hypometabolism in migraine patients with and without aura, with associations with disease duration [37–39]	• Hypoglycaemia reduced CSD threshold, increased duration and exhibited spontaneous CSD events [29–31]	Repeated migraine attacks and activation of nociceptive regions may lead to abnormalities in glucose metabolism
Lactate production	• Serum and plasma measurements exhibit upregulated lactate in migraine patients both with and without aura [44–46] • ^1^H-MRS studies demonstrated increased lactate in familial hemiplegic migraine and migraine with aura, but not in migraine without aura [48–50]	• Rat ^1^H-MRS studies exhibit increased lactate and reduced pH during CSD [41]	Lactate excess may be produced as a result of tissue hypoxia during metabolically challenging events such as CSD. Lactate may be used as alternative energy substrate and could signify an impaired astrocytic-neuronal interaction
Lipid metabolism	• Alterations cholesterol and LDL were identified in migraine patients [58–61] • Higher lipid content in serum was associated with frequency and severity of migraine attacks [59, 60, 62]	Following CSD in mice, blood metabolite measurements exhibit alterations in lipid metabolism including increased prostaglandin and anti-inflammatory lipid mediators [66]	It is unknown if hyperlipemia is a cause or effect of migraine or related to comorbidities. Associations with cholesterol and LDL may provide an explanation for the increased cardiovascular and stroke risk in migraine with aura [63, 64]
Fatty acid oxidation	• Chronic migraine patients demonstrated differentially metabolized fatty acids compared to controls, with levels correlating with depression [76] • PEA supplementation reduced headache attacks per month and pain intensity in migraine without aura, [78] and reduced pain intensity (in combination with non-steroidal anti-inflammatory analgesics) in migraine with aura [79]. Diets rich in omega 3 fatty acids reduced headache frequency) [80]		Fatty acids also play a role in modulating neuroinflammation and supplementation may aid in inflammatory aspects of migraine pathophysiology
Mitochondrial function and oxidative phosphorylation	• Migraine patients exhibit increased ADP in those with aura and FHM [87–89] and decreased phosphorylation potential in both migraine with and without aura [90–92] • Co-enzyme Q10 studies exhibit a reduction in migraine attack duration, [99] frequency [100], severity and migraine days per month [101] • Reduction in attack frequency, [104] and severity [104, 105] was also accomplished with use of riboflavin	• CSD causes a surge in ATP production followed by reduced oxidative capacity, [42, 83, 84] and decreased mitochondrial membrane potential [85]	A decreased mitochondrial activity and increased energetic demand which may underlie the inability to maintain optimal intracellular ionic milieu and the reduced threshold for migraine attack

ATP; adenosine triphosphate, CSD; Cortical spreading depression, ^1^H-MRS; proton nuclear magnetic resonance spectroscopy, LDL; low density lipoprotein, PEA; palmitoylethanolamide

### Current understanding of migraine pathogenesis

Sensitization of the trigeminovascular system and cortical hyperexcitability are two mechanisms thought to be crucial to pathophysiology of migraine.

The trigeminovascular system is composed of trigeminal sensory neurons innervating the dura mater as well as cerebral and pial blood vessels. They synapse in the pars caudalis of the ipsilateral spinal trigeminal nucleus. The main projection of these nociceptive fibres is the ventral posteromedial thalamic nucleus, which then relays to the primary sensory cortex. This system is fundamental to nociception and migraine pathophysiology [10]. Trigeminal afferents express a range of receptors, such as transient receptor potential (TRP) channels, which are targets of nociceptive and vasoactive agents able to promote allodynia and hyperalgesia and, hence, induce headache attacks [11]. An important molecule released by afferents is calcitonin gene-related peptide (CGRP) [12], a 37-amino acid peptide implicated in migraine and a target of recent effective migraine therapeutics [13–17].

Hyperexcitability of the cerebral cortex is also thought to contribute towards migraine pathophysiology [18]. It has been hypothesised that hyperexcitability of trigeminovascular neurons may account for headache during migraine attacks in the absence of aura. Hyperresponsiveness of the visual cortex may result in photophobia or of the auditory cortex which corresponds to avoidance of noise [19]. Moreover, Familial Hemiplegic migraine (FHM), features mutations in calcium, ATP or sodium channels which result in a lowered threshold for neuronal activation. One mechanism of hyperexcitability which may contribute toward headache pathophysiology is cortical spreading depression (CSD): a wave of depolarisation across the cortical surface, leading to release in neuropeptides and alterations in cerebral blood flow [20, 21]. Functional imaging studies have associated this neurophysiological event with migraine aura: [22] a period of temporary visual disturbances and focal neurological symptoms that can precede a migraine attack [23]. Mechanistic insights from animal studies suggest that CSD can also activate meningeal nociceptors and, hence, may contribute towards headache pain [24]. Cortical spreading depression is of particular importance to this review as it is a metabolically stressful event and has been hypothesised to be triggered by or lead to, an energetic imbalance in migraine patients [25].

### Dysfunctional glucose metabolism in migraine

**Fig. 1:**
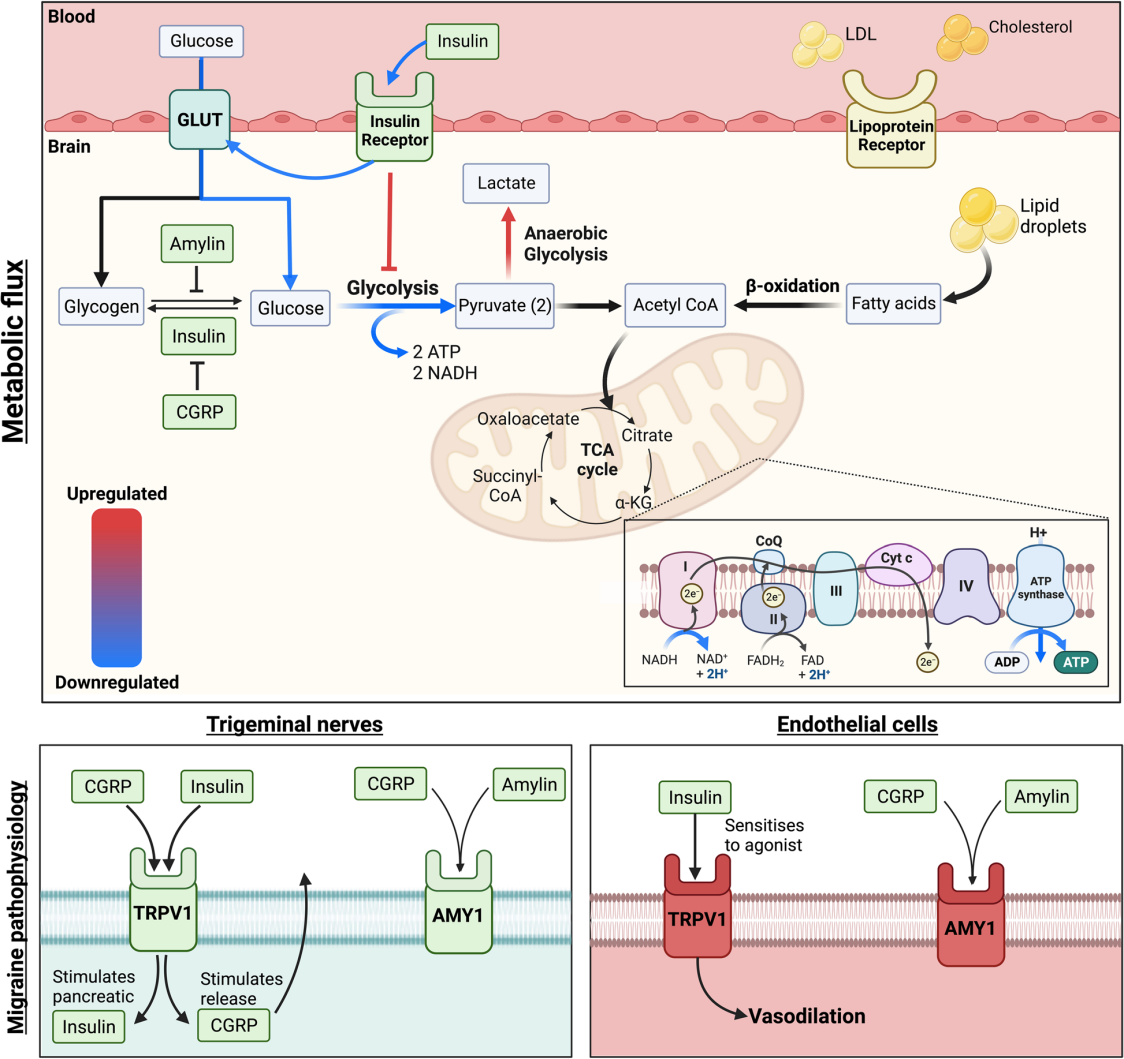
Hypothesised alterations in metabolic flux which may contribute to migraine pathophysiology. The activity of numerous vital metabolic pathways is altered in migraine patients, which may lead to the upregulation of nociceptive peptides. Moreover, metabolic hormones including insulin and amylin act on receptors implicated in nociception in trigeminal nerves and vasodilation in endothelial cells, pathways which are both involved in migraine pathophysiology. α-KG; α-ketoglutarate, ADP; Adenosine di-phosphate, AMY1; Amylin type 1 receptor, ATP; Adenosine tri-phosphate, CGRP; Calcitonin gene related peptide, CoQ; CoenzymeQ10, Cyt c; Cytochrome C, FAD; Flavin adenine dinucleotide, GLUT; Glucose transporter, TCA; tricarboxylic acid cycle

Glucose is the major energy substrate of the brain. It diffuses across the blood–brain-barrier via the glucose transporter GLUT1 and can be taken up by neurons via GLUT3 [26]. Its major metabolic pathways are: glycolysis (yielding ATP molecules as well as pyruvate or lactate), glycogenesis (providing glycogen stores which can be utilised during hypoglycaemia or ischaemia) and the pentose phosphate pathway (involved in ribose-5-phosphate and NADPH metabolism). Pyruvate can be metabolised in the tricarboxylic acid (TCA) cycle following its oxidative decarboxylation into Acetyl CoA via the highly regulated and irreversible pyruvate dehydrogenase reaction (Fig. 1).

Glucose metabolism is fine-tuned within and among cells to ensure maintenance of adequate concentrations of substrates. At rest, the metabolism of the brain is compartmentalised between neurons and astrocytes; neurons can rely on both glycolytic and oxidative metabolism, whereas astrocytes tend to be primarily glycolytic and can metabolically support neurons [27]. At times of increased energy demand, glycolysis is upregulated in neurons as the pathway can provide ATP at a faster rate than mitochondrial oxidative phosphorylation [28]. Hypoglycaemia leading to depleted glucose supply or dysfunctions in glucose uptake and glycolysis can have deleterious effects on cerebral function and have been demonstrated in migraine.

In vivo murine models have been useful in providing in-depth understanding of mechanisms linking abnormal glycolysis and migraine (Table 1). In rodents, hypoglycaemia induced by food deprivation or insulin was shown to reduce CSD threshold whilst increasing its duration [29, 30]. Spontaneous depolarisation events, which are analogous to anoxic depolarisation, have also been recorded in hypoglycaemic rats [31]. These studies propose the theory that inability to maintain ionic homeostasis, due to depleted ATP production, may increase excitability of cortical tissue and, therefore, susceptibility to CSD. In contrast, hyperglycaemia provided a protective effect, increasing the electrical threshold and reducing the frequency of potassium chloride-induced CSDs [29]. In other models however, both glucagon (increasing blood glucose) and insulin (decreasing blood glucose) inhibited dural-evoked neuronal firing in the trigeminocervical-complex [32]. These studies were very useful to determine that trigeminal nerves are glucose responsive, and suggest a role for hormones regulating metabolic homeostasis in pain (further detailed in sections below) [32].

In clinical practice, migraineurs often report that attacks are precipitated by fasting or skipping meals, [33] and indeed food deprivation was associated with headache in 58% of patients with frequent migraine or tension type headache [34]. In addition to being a migraine trigger, hypoglycaemia is thought to have a causative role in fasting headache, defined as occurring following 16 h of fasting and resolving within 72 h of food intake [23]. Another example is the GLUT1 deficiency syndrome: it results in impaired facilitated diffusion of glucose into the brain and can cause epileptic encephalopathy, however, there are milder phenotypes that demonstrate hemiplegic migraine [35, 36]. It is plausible that GLUT1 deficiency causes a reduction of the major energetic substrate of the brain and may trigger headache attacks via insufficient ATP production. Interestingly, hemiplegic migraines resolved with a modified Atkins diet which supplies ketone bodies as an alternative energy source [35]. This observation further supports the theory that metabolic imbalances are highly relevant to migraine and may be corrected by providing appropriate metabolic support.

The gold standard to assess cerebral glucose uptake in vivo in patients is 18F-Fluorodeoxyglucose PET (18F-FDG PET) which has provided further evidence for the role of downregulated glycolysis in migraine. The cerebral areas affected by glucose hypometabolism appear to be different in episodic migraineurs compared to chronic patients. In episodic migraineurs both with and without aura, hypometabolism has been detected in temporal [37], and fronto-temporal [38] regions involved in pain processing, whereas prefrontal cortex appears to be implicated in chronic patients [39]. Hypometabolism was also associated with disease duration in patients with and without aura, [37, 39] suggesting that repeated migraine attacks and activation of nociceptive regions may lead to abnormalities in glucose metabolism. Hypometabolism was improved in episodic migraine patients following external trigeminal nerve stimulation, which also decreased migraine attack frequency [38]. These studies suggest that improving glucose availability and utilisation may offer a prophylactic solution in some migraine patients and is an avenue to be explored further to expand available migraine treatment options.

### Lactate

In the absence of sufficient oxygen, lactate is produced via anaerobic glycolysis of pyruvate (Fig. 1), hence, it has often been used as a pathological marker of hypoxia. However, lactate is routinely produced by specific cells and is an important signalling molecule involved in regulating neuronal plasticity, excitation, and homeostasis [40].

Results of animal and clinical studies suggest that lactate excess may be related to CSD. This hypothesis was corroborated in rat proton magnetic resonance spectroscopy (^1^H-MRS) studies during CSD, [41] and also supported by analyses of metabolites in brain tissue which demonstrated increased lactate, reduced glucose and pH. These results suggest the occurrence of lactic acidosis in neuronal tissue, therefore it is possible that vascular changes during CSD may lead to tissue hypoxia and the preferential use of anaerobic processes [42]. Not only is anaerobic glycolysis less efficient at ATP production, but it is plausible that it may also delay the recovery period, which is particularly energy intense. Interestingly, some studies have shown that during the metabolically challenging period following a CSD, lactate is produced in excess by astrocytes and utilised as an energy substrate by neurons [43]. Although healthy neuronal tissue demonstrates plasticity to changes in energetic flux in vivo, this process may become pathological during chronification of migraine and repeated CSD events, possibly via an impaired astrocytic-neuronal interaction.

Further evidence for the role of lactate in migraine comes from studies conducted in humans (Table 1). Upregulated lactate concentrations have been shown in migraine patients both with and without aura during interictal periods in direct measurements of serum and plasma [44–46]. Interestingly, exercise can be a trigger of migraine attacks in some migraineurs, and, although the exact pathophysiology remains unknown, excessive lactate production may contribute towards aetiology. For example, in a study including 20 chronic migraine patients and 20 controls, lactate was increased in migraine in comparison to controls during aerobic exercise (although significant results did not survive correction for multiple comparisons) [47]. ^1^H-MRS studies, which allows direct detection of lactate in vivo in brain, have also demonstrated increased lactate in familial hemiplegic migraine [48] and in migraine with aura, [49, 50] although not in migraine without aura. These observations strengthen the link between CSD (which is associated to aura) and lactate in migraine [51].

### Lipid metabolism: cholesterol

Lipids can also be utilised as an energy source and are stored by astrocytes. Lipid droplets can be metabolised via fatty acid β-oxidation to produce acetyl-CoA which can then be oxidised in the TCA (Fig. 1). Fatty acids can also cross the blood–brain-barrier, and following oxidation, provide metabolic support to neurons in the form of ketones, NADH, acetyl CoA, and FADH^2^.

Dysregulation of lipid metabolism has become evident in migraine, since obesity has been associated with a higher risk, [52] increased frequency, [53] worse prognosis and chronification of migraine [54, 55]. CGRP has also demonstrated a key role in lipid metabolism and energy homeostasis, since high fat meals lead to increased plasma CGRP concentrations [56]. Moreover, plasma CGRP concentrations were increased in obese women compared to normal weight controls [56]. The expression of several neurotransmitters and oestrogen receptors in adipose tissue, [57] has led to the theory that the hypothalamic-pituitary-adipose axis may contribute towards headache pathology. It is possible that CGRP has a pivotal role in this axis. Whilst CGRP monoclonal antibodies are effective at treating migraine, their long-term effects on bodyweight are still unknown and would need further investigation.

Alterations in blood lipids has been identified in migraine patients (particularly cholesterol and low-density lipoprotein -LDL-), [58–61] with higher lipid content in serum being associated with elevated frequency and severity of attacks (Table 1) [59, 60, 62].

Importantly, such direct associations with cholesterol and LDL may provide an explanation for the increased cardiovascular and stroke risk in migraine [63, 64]. There is a significant association between stroke and migraine with aura, [64, 65] and although some studies identified stronger associations between elevated total cholesterol and triglycerides and migraine with aura, [60], others found that patients exhibited lipid alterations independent of aura symptoms [59]. Both total and LDL cholesterol were reduced in patients after therapy, [62] suggesting that perturbed lipid metabolism is reversible. It is not yet known if increased cholesterol in migraine is a cause, an effect or correlations are due to underlying common factors. However, metabolite measurements in blood from mice following CSD also demonstrate alterations in lipid metabolism including increased prostaglandin and anti-inflammatory lipid mediators [66]. Additional studies in murine model are needed to further elucidate links between migraine, aura subtypes and cholesterol metabolism as well as pathophysiology. They could translate into changes into clinical practice as lowering total cholesterol and LDL may improve migraine [62].

### Secondary headache and lipid metabolism

An association between obesity and headache is also evident in secondary headache disorders including idiopathic intracranial hypertension (IIH). IIH is characterised by raised intracranial pressure and features headache with migraine-like characteristics [67]. Over 90% of IIH patients are obese, [68] and the incidence of the disease is increasing in parallel with rising obesity rates [69]. In newly diagnosed female IIH patients, higher BMI and moderate weight gain of 5% were associated with greater risk of IIH [70]. Moreover, adiposity was found to be associated with disease activity and insulin resistance in IIH, with adipocytes being transcriptional primed for gaining adipose mass [71]. Weight loss via surgical or diet interventions is therapeutic in IIH and was associated with improvement in headache measures [72, 73]. Investigating the metabolic pathways underpinning the pathogenesis of IIH would be beneficial to development of biomarkers and therapeutic targets.

### Fatty acid metabolism

Prolonged activity neurons leads to upregulated β-oxidation and consequently increased reactive oxygen species (ROS) and peroxidated fatty acids [74, 75]. Using high throughput mass spectrometry methods, plasma fatty acids were found to be differentially metabolized in chronic migraine patients compared to controls, with levels correlating with depression [76]. Improving fatty acid metabolism may also provide therapeutic benefits. This has been suggested in preliminary studies testing the effects of supplementation with Palmitoylethanolamide (PEA), an endogenous fatty acid amide found within the central nervous system and able to stimulate fatty acid oxidation [77]. PEA supplementation reduced number of headache attacks per month and pain intensity in paediatric patients with migraine without aura, [78] whereas it reduced pain intensity (when given in combination with non-steroidal anti-inflammatory analgesics) in migraine with aura [79]. Moreover, diets rich in omega 3 fatty acids (such as containing 1.5 g of eicosapentaenoic acid and docosahexaenoic acid a day) were also found to reduce headache frequency compared with a normal intake of fatty acids (150 mg of eicosapentaenoic acid and docosahexaenoic acid a day) [80].

Fatty acids also play a role in modulating neuroinflammation, which is a function potentially of benefit in neurodegenerative diseases [81]. Hence, supplementing the diet with sources of fatty acids is a migraine treatment approach that needs exploring in further randomised trials which may show benefits in other conditions as well.

### Mitochondrial function and oxidative phosphorylation

Healthy neurons rely primarily on oxidative phosphorylation for energy, a process hosted by mitochondria which are vital organelles.

For the investigation of mitochondrial function in vivo, most models utilise CSD as a mechanism of migraine. Use of two-photon fluorescent imaging to measure the ratio of autofluorescent reduced NADH to non-fluorescent NAD^+^ allows quantification of changes in mitochondrial redox potential [82]. Using this method, it has been possible to observe an initial surge in NADH oxidation and ATP production followed by a prolonged period of depleted oxidation [42, 83, 84]. It is plausible that this prolonged period of reduced NADH utilisation may be due to the limited supply of oxygen in neural tissue due to vascular changes during CSD [42]. Moreover, repetitive CSD events were able to gradually decrease baseline fluorescent changes, suggesting that chronic CSD events may lead to long-term alterations in the oxidative capacity of the tissue [84]. In addition to reduced oxidative capacity, CSD also resulted in a decreased mitochondrial membrane potential and therefore activity of the ATP synthase in rats [85]. Dural exposure to inflammatory soup resulted in a reduced spare respiratory capacity, specifically in the trigeminal nucleus caudalis in acute brain slices [86]. In these models, reduced mitochondrial function not only resulted in depleted ATP supply, but also in increased production of reactive oxygen species and calcium influx, which may all contribute towards the pathology of headache besides altering metabolic flux.

The modality of choice to investigate mitochondrial function in vivo in humans is 31-Phosphorus magnetic resonance spectroscopy (^31^P-MRS). Studies in migraine have consistently highlighted primary mitochondrial dysfunction in patients as evidenced by increased ADP in those with aura and FHM [87–89] and decreased phosphorylation potential (indicating reduced available free energy) in both patients with and without aura (Table 1) [90–92]. Together these results are consistent with those reported in ^31^P-MRS studies of mitochondrial cytopathies and some neurodegenerative conditions such as amyotrophic lateral sclerosis [93, 94]. Results suggest an imbalance between energy demand and production which may be the underlying reason for inability to maintain optimal intracellular ionic milieu and the reduced threshold for migraine attack. ^31^P-MRS data in patients may reciprocate findings in CSD animal models, since migraine with aura patients most frequently demonstrate alterations in mitochondrial function. Results regarding changes in ATP concentrations remain divided, with most studies either not reporting ATP levels, or concentrations remaining unchanged or similar to that of controls [95]. ATP may remain constant whilst free energy is reduced since depleted ATP is demonstrated mostly in necrotic tissue, [96, 97] which is not typically a feature of migraine. Some further indirect evidence was provided by analysis of the transcriptome of peripheral blood mononuclear cells from migraine patients, which also revealed alteration in pathways linked to oxidative phosphorylation, with the majority of altered genes being downregulated compared to controls [46].

Lastly, a strong argument for dysfunction in oxidative phosphorylation in migraine is provided by the therapeutic benefits of coenzyme Q10 (CoQ10) supplementation. CoQ10 is an electron acceptor in the electron transport chain which has been shown to improve mitochondrial respiration in mitochondrial cytopathies [98]. Several trials have demonstrated the ability of CoQ10 to reduce migraine attack duration, [99] frequency, [100] severity and migraine days per month [101]. In addition to acting in the electron transfer chain, CoQ10 also has an anti-inflammatory role which may be favourable in the treatment of migraine, and was able to reduce nitric oxide (NO), [102] TNF-α and CGRP in a placebo-controlled trial [103]. Reduction in attack frequency, [104] and severity [104, 105] was also accomplished with use of riboflavin further supporting the role of dysfunctional oxidative phosphorylation in migraine. Riboflavin is a precursor of both FAD flavin adenine dinucleotide (FAD) an electron donor involved in complex II and flavin mononucleotide (FMN), a component of complex I.

### Insulin

Insulin has become a hormone of interest in migraine since numerous studies have identified insulin resistance in patients [106–109]. Resistance was also associated with migraine disease duration in a study which reported metabolic syndrome in 31.9% of chronic migraine patients [109]. Although there were no differences found between those with or without aura, identification of a single nucleotide polymorphism in the insulin receptor gene has been found to be associated with migraine aura, further implicating the role of insulin function in hyperexcitability involved in migraine aetiology [110]. However, until studies are conducted comparing insulin function in migraine patients with obesity or diabetes versus obese or diabetes controls, it remains difficult to attribute changes in insulin resistance solely to migraine.

Animal studies have revealed that insulin may potentially modulate the release of CGRP. In particular, insulin can induce the release of CGRP via sensitization of neuronal Transient Receptor Potential Cation Channel Subfamily V Member 1 (TRVP1—Fig. 1) [111]. Insulin is also able to sensitize vascular TRPV1 receptors to induce vasodilation, a similar effect shown in isolated mesenteric blood vessels of rats [112]. In addition to their activation, vascular TRPV1 receptors are also sensitized to its agonists following insulin interaction, inducing a more pronounced vasoconstrictive effect when activated with capsaicin [111].

CGRP is also able to regulate insulin secretion [113] via TRPV1 activation, [114] potentially suggesting a feedback loop between excessive nociception and altered insulin function. TRPV1 knockout mice demonstrated glucose intolerance, whereas agonism of TRVP1 in wild type mice induced insulin secretion [114]. Moreover, CGRP infusions in rat and dog models have exhibited multiple markers of insulin resistance including increased plasma glucose, [115] decreased glucose uptake, [116] impaired glycogen synthesis in muscle, [117] and increased hepatic glucose production [116]. Ex vivo investigation of pancreatic islets in a model of diabetes and diet-induced obesity exhibited CGRP’s ability to block glucose-stimulated insulin secretion, insulin-2 gene expression and reduce glycolytic capacity [118]. One may speculate as to whether insulin dysfunction may be a predisposing feature of metabolic abnormalities in migraine, or if it is a result of chronic CGRP exposure. Investigating the effects of disturbed insulin secretion such as in type-2 diabetes patients on CGRP concentrations and migraine prevalence would provide further evidence of a reciprocal relationship.

Although antagonism of CGRP signalling has demonstrated significant efficacy in migraine prophylaxis, the effects on insulin function have not yet been assessed in patients. CGRP receptor antagonism in mice has shown moderate improvements in oral glucose tolerance [119]. CGRP-α knockout mice exhibited improved insulin sensitivity and glucose handling in response to a high-fat diet [120]. In other models of diabetes and diet-induced obesity, the use of antibodies to block CGRP-α also improved glucose tolerance, insulin sensitivity and resulted in weight loss [118]. The α isoform of CGRP may have a more dominant role in glucose handling, since inhibition of both α and β isoforms upregulated insulin secretion but did not affect plasma glucose concentrations [121]. Since CGRP antagonism has shown promising results at improving glucose and insulin metabolism in vivo, it would be important to probe putative benefits in metabolism in patients with migraine.

### Amylin

Amylin is a pancreatic hormone co-released with insulin in response to food intake [122]. It lowers serum glucose by reducing glycogen release and may have a direct role in trigeminal nerve sensitisation, since it shares a substantial amino acid similarity to CGRP [123]. Both amylin and CGRP can bind to theAMY1 receptor which is located throughout the trigeminovascular system [124]. It has also been shown to be present in elevated concentrations in migraine patients [125]. Moreover, a recent provocation study demonstrated that infusion of the amylin analogue Pramlintide, induced migraine attack in migraine without aura patients [126]. Similar studies in mice also indicated sensitisation following amylin infusion by decreasing Von Frey thresholds and increasing aversion of bright light, particularly in female mice [126]. Squint-detecting assays, used as an automated measure of grimacing and pain, also detected increased squinting in female mice following amylin injection [127].

Whilst CGRP has become a popular target for migraine therapeutics, the therapeutic potentials of amylin antagonism have not yet been fully explored. Pramlintide, an amylin receptor agonist, is currently licensed for diabetes in the United States [128]. It may be important to consider headache as a side effect in diabetic patients, since its off-target effects may include activation of the trigeminovascular system.

## Conclusion

The pathophysiology of migraine is evolving and may also feature mitochondrial and metabolic deficits. Moreover, migraine is prevalent in those with mitochondrial disorders and structural and biochemical impairments in electron transfer chain have been identified in migraineurs [129]. Interesting, CGRP a major nociceptive peptide involved in migraine demonstrates a reciprocal relationship with multiple metabolic pathways including glucose and lipid utilisation. It is still uncertain as to whether metabolic deficits result in excessive CGRP-mediated nociception or vice versa. Attributing metabolic perturbations exclusively to migraine may be difficult, since common migraine comorbidities also feature some metabolic alterations such as obesity and depression [130]. However, both clinical and in vivo evidence suggests that an imbalance between energetic demand and supply may contribute towards migraine pathology. Therefore, perturbation of metabolic pathways which exacerbate this imbalance may be the basis for the metabolic component of migraine.

## Data Availability

Not applicable.

## Abbreviations

^31^P-MRS: Phosphorus magnetic resonance spectroscopy
18F: Fluorodeoxyglucose PET (18F-FDG PET)
ADP: Adenosine diphosphate
AMY1: Amylin type 1
ATP: Adenosine triphosphate
CGRP: Calcitonin gene related peptide
CoQ10: Coenzyme Q10
CSD: Cortical spreading depression
FADH^2^: Flavin adenine dinucleotide
GLUT: Glucose transporter
LDL: Low density lipoproteins
MRS: Magnetic resonance spectroscopy
NADH: Nicotinamide adenine dinucleotide
TNF-α: Tumour necrosis factor alpha
TRP: Transient receptor potential
TRPV1: Transient Receptor Potential Cation Channel Subfamily V Member 1
TCA: Tricarboxylic acid
